# Microfluidic Fibroblast Cell Culture Chip for Embryo Co-Culture: Analysis of Preimplantation Embryo Viability and Development Potential

**DOI:** 10.3390/ijms27156592

**Published:** 2026-07-24

**Authors:** Ya-Shun Lo, Tian-Chi Tsai, Te-Yu Tsou, Kai-Cheng Chang, Yi-Wen Wang, Wen-Syang Hsu, Da-Jeng Yao, Hong-Yuan Huang

**Affiliations:** 1 Department of Obstetrics and Gynecology, Chang Gung Memorial Hospital, Linkou Medical Center, 5, Fu-Shin Street, Taoyuan 33301, Taiwan; lavendaaaa@cgmh.org.tw (Y.-S.L.); mp2186@cgmh.org.tw (T.-C.T.); iwen0711@cgmh.org.tw (Y.-W.W.); 2 Institute of NanoEngineering and MicroSystems, National Tsing Hua University, Hsinchu 30013, Taiwan; skps940063@gmail.com (T.-Y.T.); kenchang9458@gmail.com (K.-C.C.); 3 Department of Mechanical Engineering, National Yang Ming Chiao Tung University, Hsinchu 30010, Taiwan; whsu@nycu.edu.tw; 4 Department of Obstetrics and Gynecology, College of Medicine, Chang Gung University, Taoyuan 33305, Taiwan

**Keywords:** cell coculture, embryo, endometrium, microfluidic, organ-on-a-chip

## Abstract

Preimplantation embryo development requires a tightly regulated microenvironment that is not fully reproduced by conventional static culture. We developed a polydimethylsiloxane-based microfluidic embryo co-culture platform integrating compartmentalized architecture with dynamic perfusion to simulate physiological conditions. The study included two stages. First, NIH/3T3 mouse fibroblasts were evaluated as helper cells under three culture conditions after transition to embryo culture medium. Helper-cell viability in the dynamic chip was 84.72%, compared with 71.91% after manual medium replacement in 24-well plates and 94.27% in the 24-well control group. Second, mouse embryos were cultured under four conditions: conventional 24-well plates, static microfluidic chip culture with co-culture, dynamic microfluidic chips without co-culture, and dynamic microfluidic chip co-culture. Blastocyst formation rates were 100.0% (9/9), 0.0% (0/6), 33.3% (3/9), and 55.5% (5/9), respectively. Because no inferential statistical analysis was performed, these proportions are interpreted descriptively. Nevertheless, the blastocyst formation in the microfluidic co-culture group supports the technical feasibility of integrating dynamic perfusion and helper-cell co-culture within a single platform. Further optimization and validation are required. This platform provides a foundation for future development of advanced embryo culture technologies including patient-specific endometrial co-culture systems in assisted reproduction, disease modeling, or drug development.

## 1. Introduction

Embryo development and implantation depend on precise molecular, cellular, and biomechanical interactions within the female reproductive tract. In vivo, early embryos develop in a dynamic microenvironment where surrounding tissues continuously provide nutrients, growth factors, and antioxidants, while metabolic by-products are efficiently removed through fluid movement [1]. Conventional in vitro embryo culture systems are predominantly based on static platforms with limited fluid exchange. Although such systems support embryonic development, they do not fully recapitulate the dynamic transport of signaling molecules and metabolic substrates that characterizes the in vivo reproductive tract. Accumulation of metabolic by-products and oxidative stress may alter the embryo microenvironment, potentially influencing developmental competence [2,3,4].

Microphysiologic systems (MPS), including organ-on-a-chip and microfluidic platforms, have emerged as powerful tools for modeling complex biological microenvironments in vitro [5,6]. By integrating microfluidic flow with three-dimensional cell culture, these systems enable precise control of biochemical gradients, mechanical cues, and cell–cell communication [6,7]. Recent advances in reproductive organ-on-a-chip models have demonstrated the feasibility of reconstructing hormonally responsive endometrial microenvironments, supporting sex steroid-dependent cellular responses and stromal–epithelial interactions [8,9,10,11]. However, applications of MPS to preimplantation embryo culture with endometrial cell co-culture remain underdeveloped.

Given the dynamic nature of embryo–maternal communication in vivo, the integration of controlled microfluidic flow and helper-cell co-culture for embryo development represents a promising strategy for studying embryo development under controlled medium exchange and helper-cell co-culture. Dynamic perfusion may facilitate sustained interactions mediated by factors released by supporting cells while limiting the accumulation of metabolic waste and oxidative by-products [4,12,13]. Animal studies have demonstrated that such microenvironments can support embryonic development comparable to or better than static systems [14,15]. However, human randomized controlled trials evaluating dynamic culture systems without co-culture have not demonstrated consistent clinical benefits [16], suggesting that additional factors—such as the integration of helper-cell co-culture—may be necessary to realize the potential advantages of microfluidic platforms.

In this study, we developed a three-dimensional biomimetic microfluidic embryo culture platform designed to model key structural and functional features of the uterine microenvironment [1,17,18]. By combining compartmentalized co-culture architecture with controlled fluid dynamics, this system aims to recapitulate molecular signaling and biomechanical cues relevant to embryo–endometrium communication. We hypothesized that microfluidic co-culture with fibroblast helper cells would improve preimplantation embryo viability and developmental potential compared to static culture.

## 2. Results

### 2.1. Microfluidic Chip Design and Operation

#### Chip Geometry and Structural Design

**Figure 1:**
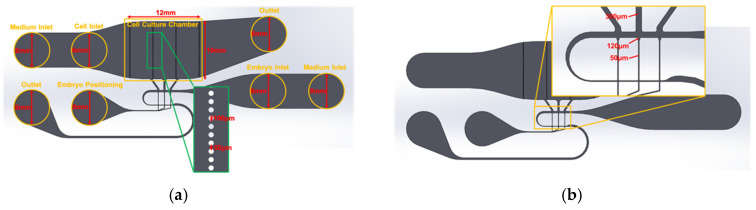
Microfluidic chip design. (**a**) Overall layout of the chip, showing the upper co-culture chamber and the lower embryo loading and transport region. The co-culture chamber measures 12 mm × 10 mm and is divided into three compartments connected by microchannels with a width of 100 μm. All inlet and outlet ports are 6 mm in diameter. (**b**) Enlarged view of the lower layer for embryo loading and trapping structure. Key channel widths are 300 μm, 50 μm and 120 μm.

The microfluidic chip consisted of two functional layers: an upper co-culture chamber and a lower embryo handling region. The upper layer served as the main chamber. It measured 12 mm × 10 mm and was divided into three compartments connected by 100 μm microchannels, which allows medium exchange and communication between compartments while maintaining a compartmentalized culture architecture. The upper layer contained inlet ports for culture medium infusion and cell suspension introduction, as well as an outlet port. The lower layer contained ports for embryo loading and positioning. All inlet and outlet ports had an outer diameter of 6 mm (Figure 1a).

To confine embryos within the designated culture chamber during cultivation, flow-resistance structures were incorporated at the chamber boundaries in the lower layer as shown in Figure 1b [19]. Given that the average diameter of mouse embryos is approximately 100 to 150 μm [20], the connecting microchannels between the culture chamber and adjacent regions were designed with widths smaller than 100 μm. This configuration prevented embryo displacement while still permitting medium perfusion and removal of cellular debris and metabolic by-products.

The chip was fabricated using a photolithographic molding process with a photoresist spin-coating speed of 1000 rpm, resulting in an average channel height of approximately 220 μm. The culture chamber had an average surface area of 201.06 mm^2^ and an average volume of 44.23 μL, while the total chip volume was approximately 74.13 μL.

### 2.2. Static Culture of Supporting Cells on the Microfluidic Chip

Mouse fibroblast cells (National institutes of health/3-day transfer, NIH/3T3), which are commonly employed in microfluidic validation studies [21], were applied in our study. They were first assessed on cell attachment, proliferation, and stability within the microfluidic chip under static culture conditions. Different seeding densities were tested to determine a suitable condition for co-culture experiments. Cell morphology and surface coverage were recorded every 24 h, with medium replacement performed at the same interval. Seeding at 3 × 10^6^ cells achieved over 80% coverage within 48 h. At lower seeding density, cells proliferated gradually and reached approximately 80% surface coverage only after 72 h. Therefore, we opted for 3 × 10^6^ cells/mL medium for subsequent experiments. At this higher density, partial cell detachment and floating cells were observed by 72 h, indicating that more frequent medium replacement was required to maintain culture stability [22].

### 2.3. Dynamic Culture of Supporting Cells Under Continuous Perfusion

After an initial attachment period of 2 h, continuous DMEM perfusion was applied at a flow rate of 0.4 μL/min, which is within the optimal range reported for fibroblast culture in microfluidic systems [23]. This flow rate did not disrupt cell attachment and enabled continuous nutrient supply and removal of metabolic waste.

**Figure 2:**
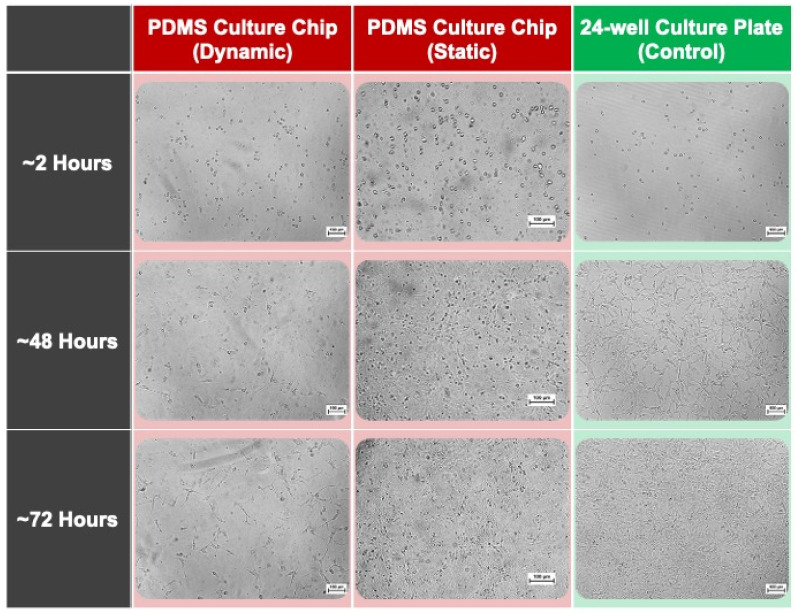
Representative morphology of co-culture cells under dynamic chip, static chip, and conventional 24-well culture conditions. At 72 h, progressive cell detachment and floating were observed in the static chip, whereas cells in the dynamic chip remained attached under continuous perfusion. Scale bar = 100 μm.

Under dynamic culture conditions, supporting cells maintained stable attachment and surface coverage, particularly at time points when static daily medium replacement was insufficient to sustain high-density cultures. Continuous perfusion supported prolonged cell maintenance within the microfluidic chip (Figure 2).

### 2.4. KSOM Transition and Supporting Cell Viability

To establish conditions compatible with embryo co-culture, culture media were transitioned from DMEM to KSOM, a medium specifically optimized for preimplantation embryo development [24]. In 24-well plates, we noted that direct replacement with KSOM reduced supporting cell proliferation and induced morphological changes, including cell rounding, consistent with the different metabolic requirements of somatic cells compared to embryos. When medium replacement was performed after 48 h of initial culture, cells exhibited improved stability and maintained higher surface coverage.

The same protocol was applied in the microfluidic chip. Supporting cells were cultured for 48 h until surface coverage exceeded 80%, followed by KSOM replacement at a flow rate of 10 μL/min for a total volume of 100 μL. Cells maintained comparable surface coverage following KSOM transition.

**Figure 3:**
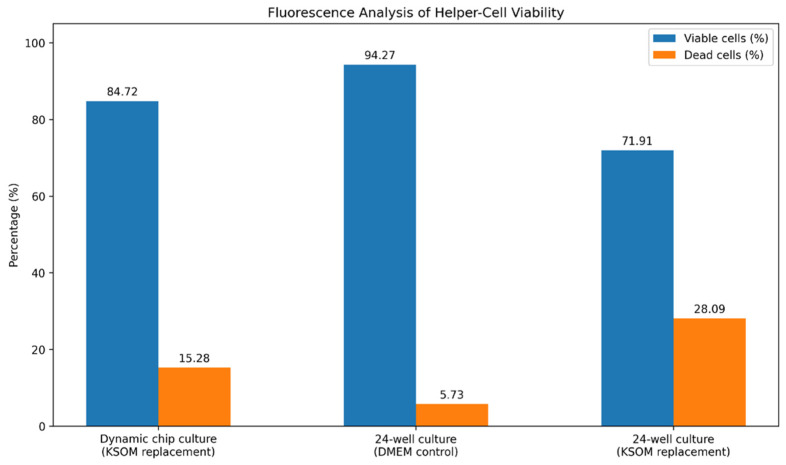
Fluorescence analysis of helper-cell viability under different culture conditions. Helper-cell viability was assessed in dynamic chip culture after KSOM replacement and compared with 24-well DMEM control and 24-well culture after manual KSOM replacement.

Cell viability was evaluated using LIVE/DEAD fluorescence staining after 48 h of KSOM exposure. Supporting cells cultured dynamically in the microfluidic chip exhibited a viability of 84.72%. In comparison, cells cultured in 24-well plates showed a viability of 94.27% under continuous DMEM conditions and 71.91% following manual KSOM replacement (Figure 3). The improved viability in the microfluidic system may be attributed to continuous nutrient supply and removal of metabolic waste during dynamic perfusion [25].

### 2.5. Embryo Co-Culture Under Static and Dynamic Conditions

Mouse embryos at the 2-cell stage were cultured under four conditions: conventional 24-well plate culture (*n* = 9 embryos), static microfluidic chip culture with co-culture (*n* = 6 embryos), dynamic microfluidic chip without co-culture (*n* = 9 embryos) and dynamic microfluidic chip co-culture with supporting cells (*n* = 9 embryos). Embryo developmental stages were recorded at 24 h intervals.

**Figure A1:**
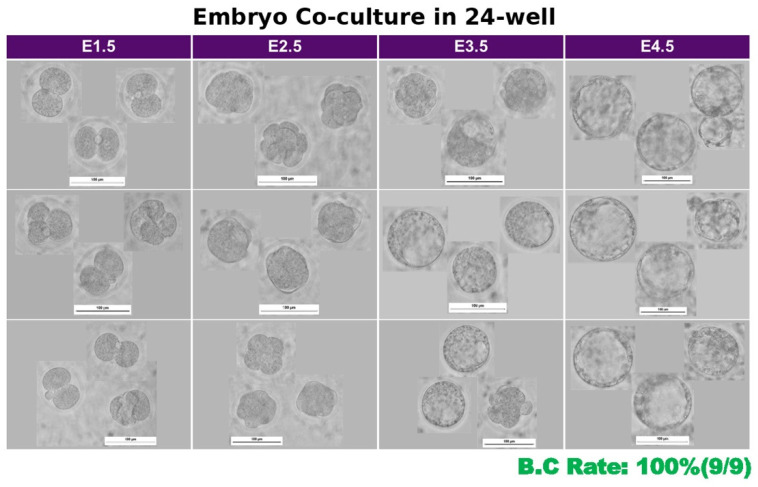
Full developmental trajectories of embryos cultured in the 24-well plate co-culture group. Embryos were followed from E1.5 to E4.5. M. indicates morula and B.C indicates blastocyst. Earlier cleavage-stage embryos were labeled according to cell number where applicable. All embryos reached the blastocyst stage by E4.5, corresponding to a blastocyst formation rate of 100.0% (9/9). Scale bar = 100 μm.

**Figure A2:**
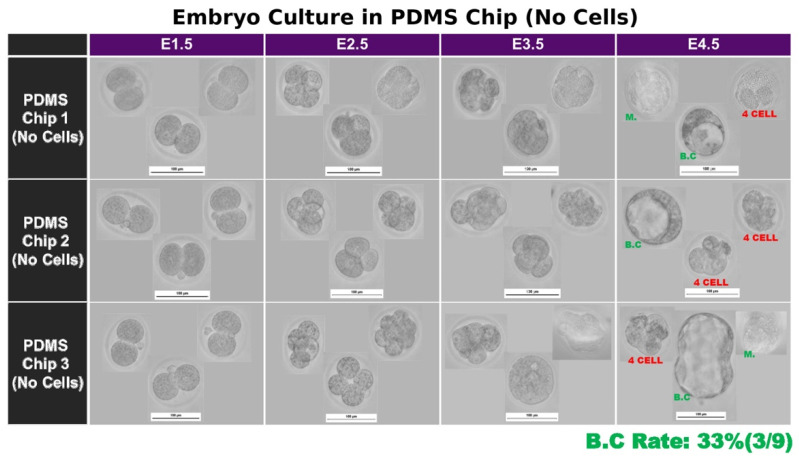
Full developmental trajectories of embryos cultured in the dynamic microfluidic group without co-culture. Embryos were followed from E1.5 to E4.5. M. indicates morula and B.C indicates blastocyst. Earlier cleavage-stage embryos were labeled according to cell number where applicable. The blastocyst formation rate was 33.3% (3/9). Scale bar = 100 μm.

**Figure A3:**
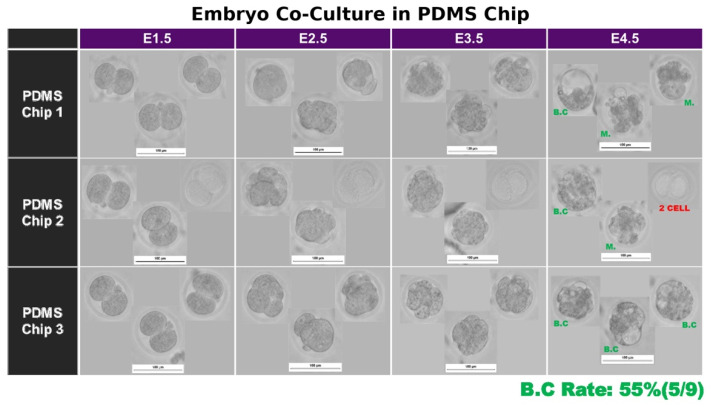
Full developmental trajectories of embryos cultured in the dynamic microfluidic co-culture group. Embryos were followed from E1.5 to E4.5. M. indicates morula and B.C indicates blastocyst. Earlier cleavage-stage embryos were labeled according to cell number where applicable. The blastocyst formation rate was 55.6% (5/9). Scale bar = 100 μm.

Across all groups, embryos progressed through cleavage stages with comparable developmental timing (Table 1). In the 24-well plate group, 100.0% (9/9) of embryos consistently developed to the blastocyst stage within 72 h (Figure A1). In the dynamic microfluidic co-culture group, 55.5% (5/9) of embryos reached the blastocyst stage (Figure A2), while 11.1% (1/9) of the embryos arrested at earlier cleavage stages and 33.3% (3/9) at the morula stage. For dynamic microfluidic without co-culture group, blastocyst-stage embryos accounted for 33.3% (3/9) (Figure A3). Static PDMS chip culture with co-culture showed lower developmental progression compared with dynamic conditions and 0.0% (0/6) of embryos progressed to the blastocyst stage. This may suggest that dynamic microfluidic chip improved the culture setting limitation through supplying nutrition and removing metabolic waste, hence gaining better embryo developing outcome than static chip culture. Full-image montages of all embryos are provided in Appendix A.

## 3. Discussion

In mammalian embryos, a series of highly orchestrated events must be achieved to reach the blastocyst stage. Following fertilization, the zygote undergoes cleavage, a series of rapid mitotic divisions within the zona pellucida [26]. Subsequently, blastulation follows, requiring precise embryonic genome activation, compaction, and fluid cavitation, which is highly energy dependent [27]. Consequently, achieving blastocyst formation in vitro is commonly used as a morphology-based developmental endpoint and an important functional indicator of a supportive culture environment [28,29,30].

In this study, our novel microfluidic co-culture chip prototype demonstrated its technical feasibility by successfully providing the complex physiological requirements necessary for embryos to reach this critical developmental milestone. Through functional integration of single-embryo tracking, automatic trapping, positioning, and controlled dynamic culture, this platform enables embryo handling and development within a microenvironment aiming to simulate the uterus. However, microfluidic embryo culturing systems, including previously demonstrated ones, have resulted in inconsistent developmental efficacy across studies [31]. This variability in developmental outcomes can be explained by several factors, including various dynamic flow rates, medium-refreshment strategies, device design and the type of co-culture cells used. Previous reports have shown that continuous medium perfusion may impair embryo development by disturbing the local embryo microenvironment, increasing shear-related stress, and removing growth-promoting autocrine/paracrine factors [32,33]. In contrast, Huang et al. demonstrated that punctual medium refreshment, pulsatile medium delivery, or periodic droplet actuation have been associated with improved embryo development [14]. These findings suggested that the optimal pattern of fluid exchange and delivery remains a challenge in embryo culture.

Our study results demonstrated a gradient in blastocyst formation rate: 100% in conventional 24-well culture plates, 55.5% in dynamic microfluidic co-culture, and 0% in static microfluidic co-culture. Because of the small sample size and absence of inferential statistical testing, these observations do not establish meaningful differences among the culture conditions. This pattern may be consistent with the hypothesis that dynamic perfusion partially mitigates the limitations of confined static microfluidic culture by facilitating nutrient exchange and the removal of toxic metabolites. However, the numerically lower blastocyst formation rate observed relative to conventional 24-well culture highlights the need for further technical and biological optimization.

One of the limitations in the dynamic system is the need to balance medium exchange with mechanical stress. It has been shown that shear stress in dynamic fluid exchange can cause embryo damage [34]. Specifically, shear stress as low as 1.2 dynes/cm^2^ can cause embryo lethality through stress-activated protein kinase-mediated apoptosis [35]. Conversely, insufficient medium turnover in a confined microfluidic chamber may increase susceptibility to metabolic waste accumulation. When metabolic waste adds up to embryotoxic levels, it prevents progression to the blastocyst stage. Ammonium accumulates linearly over time in static culture systems through spontaneous deamination of amino acids and embryo metabolism [36]. Cleavage-stage embryos are particularly sensitive to ammonium exposure, which reduces blastocyst cell number, increases apoptosis, and impairs metabolic function [37]. Future optimization should aim to improve nutrient supply and waste clearance while minimizing stress exposure.

In the present study, shear stress was not quantified by computational fluid dynamics analysis or direct experimental flow measurement. Therefore, the exact shear stress values generated within the embryo-containing regions cannot be reported, and whether the local shear environment was physiologically relevant cannot be definitively determined. Although low-flow perfusion at 0.1 μL/min was selected empirically to reduce visible embryo displacement during embryo co-culture, local shear stress may vary according to chamber geometry, inlet flow distribution, and embryo position. Therefore, the nominal flow rate should not be directly interpreted as the shear stress experienced by the embryos. Several practical strategies may be considered. First, chamber geometry should be optimized so that embryos are positioned within low-shear regions of the culture chamber, away from chamber walls and inlet jets, where local shear may be increased [38]. Second, the perfusion rate and medium exchange schedule should be optimized together. A modest increase in perfusion rate, such as 0.2–0.3 μL/min, may shorten chamber turnover time and improve waste clearance while maintaining shear stress below reported embryotoxicity threshold [35]. Alternatively, intermittent or pulsatile perfusion may be used instead of continuous perfusion to provide periodic nutrient replenishment and waste removal while reducing cumulative shear exposure [34]. However, the pulse duration, interval and flow rate should be empirically determined to avoid insufficient medium turnover or excessive shear stress. Third, future device designs may consider incorporating flow-bypass structures, allowing culture medium to perfuse through adjacent channels rather than directly across the embryo-containing region. In this configuration, embryos can remain stationary within the culture chamber while dynamic medium exchange is maintained. Future optimization should include computational fluid dynamics modeling and/or experimental flow characterization to quantify local shear stress and determine whether the embryo-containing regions remain within a physiologically acceptable shear range.

Another factor that may have influenced embryo development in the microfluidic groups is the use of PDMS for chip fabrication; however, its contribution to the observed outcomes cannot be determined from the present study. Although PDMS, a crosslinked polymer of hydrophobic dimethylsiloxane oligomers, is widely used in co-culture systems, several PDMS-related concerns have been reported. These include altering embryo metabolome and causing acute toxicity by its uncured oligomers and sequestering small hydrophobic molecules such as estrogen and progesterone through its hydrophobic network [39,40]. Estrogen and progesterone are two important molecules for preimplantation embryos; thus, the absorption may potentially interfere with embryo development, as a previous study reported 0% blastocyst formation due to this sequestration effect on mouse embryo development [41,42]. To address these PDMS-associated limitations, alternative material or PDMS pre-treatment can be considered. Polycarbonate (PC) and cyclic olefin copolymer (COC) with ultraviolet ozone pre-treatment are particularly practical candidates for embryo culture microfluidic device. Compared with PDMS, these thermoplastic materials exhibit reduced absorption of steroid hormones, optical transparency to support cell culture application, and are compatible with scalable manufacturing [43]. In addition, they may avoid PDMS-specific concerns related to uncured oligomer leaching [43,44]. For PDMS-based prototypes, Pluronic F127 pre-treatment may serve as a practical short-term strategy to reduce hydrophobic sequestration. A previous study has shown that Pluronic F127-treated PDMS supports embryo development rates comparable to polystyrene control [42]. Therefore, PC or COC with appropriate surface treatment, or Pluronic F127 pre-treatment of PDMS, may improve material compatibility in future embryo co-culture microfluidic platforms. In addition, the physicochemical microenvironment within the chip was not directly characterized. Protein adsorption, nutrient depletion, oxygen gradients, and metabolite accumulation were not quantified in the present study. Therefore, we cannot determine whether, or to what extent, PDMS-associated adsorption influenced the embryo culture microenvironment and development outcomes. Although helper-cell viability and embryo developmental progression provide functional readouts of culture performance, these outcomes cannot substitute direct physicochemical characterization. Future studies may include direct microenvironmental assessment to clarify how material properties, confined culture volume, and perfusion conditions influence embryo development.

Another important consideration is the choice of helper cells for co-culture. Primary endometrial or oviductal cells may serve as more physiologically relevant helper-cell candidates because they more closely resemble the native embryo-reproductive tract microenvironment. Primary endometrial stromal cells respond to estradiol and progesterone and participate in decidualization and implantation-associated paracrine signaling [45], whereas oviductal epithelial cells provide metabolic substrates and bioactive factors that support fertilization and early embryo development [46]. However, these reproductive tract-derived cells are technically challenging to establish and maintain reproducibly in a microfluidic device, particularly during early-stage platform development. Primary endometrial stromal cells, or oviductal epithelial cells, require technically demanding isolation procedures, careful timing according to reproductive stage [47,48], and validation of hormone responsiveness [49]. Furthermore, primary endometrial stromal cells exhibit inter-individual and batch-to-batch variability, and more complex culture conditions with hormone supplement [47]. Oviductal cells present even more technical challenges with maintenance of cell type heterogeneity to function properly [46]. These factors introduce variability and quality control challenges during early-stage microfluidic platform development. Therefore, NIH/3T3 fibroblasts were selected in the present study as a standardized and reproducible helper-cell model for proof-of-concept validation, rather than as a physiological substitute for reproductive tract-derived cells.

Although embryo co-culture with oviductal or endometrial cells may more closely resemble the in vivo reproductive tract environment, previous studies have suggested that the beneficial effects of co-culture are not strictly limited to reproductive tract-derived cells or species-matched cells [50,51,52,53]. For instance, Menezo et al. utilized VERO cells, an African green monkey kidney epithelial cell line, for human embryo co-culture and reported improved development of low-quality human embryos, with increased blastocyst formation and progression to the expanding or hatching stages [25]. Carvalho et al. found no major transcriptomic differences between bovine embryos cultured with bovine oviduct epithelial cells or VERO feeder cells, indicating that both feeder-cell types appeared to regulate similar cytokines- and growth factors-related pathways [54]. Similarly, Bagheri et al. reported that human endometrial mesenchymal stem cells supported mouse germinal vesicle oocyte maturation in heterologous co-culture systems [55]. These findings support the concept that helper-cell effects may arise from general microenvironmental support, such as secretion of embryotrophic factors, modulation of the culture environment, or removal or buffering of undesirable compounds, rather than from strict reproductive tract or species specificity [52,53].

In our study, NIH/3T3 cells were used because of their well-characterized properties, including the metabolic activity, paracrine secretion and consistent behavior across passages, making them suitable for initial proof-of-concept system development [56,57,58]. A previous study showed that when growth arrested, 3T3 cells maintain metabolic function while ceasing proliferation, allowing sustained paracrine support without overgrowing in the confined microfluidic environment [59]. These characteristics allowed optimization of technical parameters such as flow rate, shear stress, chamber geometry, medium transition, and dynamic perfusion before incorporating more physiologically relevant but technically demanding reproductive tract-derived cells. A previous study reported that co-culture with mouse embryonic fibroblasts showed improved outcomes with a 95.1% blastocyst formation rate versus the control group with 72.2%, along with increased inner cell mass cell numbers [60]. Nevertheless, NIH/3T3 cells remain a simplified biological model. They do not fully reproduce the cellular diversity, hormonal responsiveness, or secretory activity of endometrial or oviductal cells in the native reproductive tract. This limited biological fidelity may have contributed, at least in part, to the lower blastocyst formation rate observed in the dynamic microfluidic co-culture group compared with conventional 24-well culture. Future studies should compare NIH/3T3-based co-culture with primary endometrial or oviductal cell-based systems to improve the biological fidelity of future microfluidic embryo co-culture platforms.

Taken together, shear-related mechanical stress, suboptimal medium turnover in the confined culture chamber, PDMS-associated material limitations, and the limited biological fidelity of NIH/3T3 fibroblasts represent possible contributors to the lower blastocyst formation rate in the dynamic microfluidic co-culture group compared to conventional culture, as well as the absence of blastocyst formation in the static chip group. These findings demonstrate the technical feasibility of the platform rather than the superiority over conventional culture. Dynamic perfusion nevertheless remains a potentially valuable design feature because it enables controlled medium exchange within a compartmentalized embryo co-culture system.

Beyond its present role as a proof-of-concept embryo co-culture platform, this microfluidic chip may also provide a foundation for a more advanced organ-on-chip applications [9,10]. Future development of this platform may proceed in three major directions: patient-specific endometrial co-culture, disease modeling, and reproductive drug testing.

First, integration with induced pluripotent stem cell (iPSC)-derived endometrial cells could enable patient-specific embryo culture systems. The iPSC technology offers a renewable, genetically defined cell source that can be differentiated into functional endometrial stromal fibroblasts capable of hormone-responsive decidualization. Miyazaki et al. demonstrated successful differentiation of human iPSCs into progesterone-responsive endometrial stromal fibroblasts [61]. Cheung et al. further advanced this approach by developing pluripotent stem cell-derived endometrial stromal fibroblasts that exhibited cyclic hormone responsiveness when co-cultured with epithelial cells. This enabled the study of stromal–epithelial interactions crucial for implantation [62]. Campo et al. emphasized that combining iPSCs with microfluidic platforms may help mirror the complexity of endometrial biology and improve mechanistic understanding [63].

Second, microfluidic platforms have emerged as powerful tools for modeling gynecological diseases and embryo–endometrium interactions. Murphy et al. emphasized that appropriate model systems closely mimicking the architecture and function of the endometrium and its diseases are needed for conditions including endometriosis, adenomyosis, endometrial cancer, and Asherman syndrome [64]. The controlled microenvironment of microfluidic systems enables precise manipulation of hormonal, inflammatory and mechanical cues relevant to disease pathogenesis [65]. Thus, with further incorporation, our platform could be expanded from an embryo culture chip into a disease-relevant implantation model.

Third, the compartmentalized architecture of our platform may provide a framework for studying implantation dynamics and reproductive toxicology. For example, Ahn et al. demonstrated a three-dimensional vascularized endometrium-on-a-chip that recapitulated hormonal responses, provided proof-of-concept for embryo implantation modeling. It also enabled reproductive drug testing by evaluating levonorgestrel-induced changes in endometrial permeability and vascular regression [11]. Our system’s ability to maintain controlled perfusion while supporting cell co-culture positions it as a suitable foundation for such advanced modeling approaches. Regarding drug discovery and toxicology, the FDA Modernization Act 2.0 allows for alternatives to animal testing for generating preclinical data, which could significantly impact the development of therapeutics for use during pregnancy. Ingber noted that human organ chips can recapitulate organ-level physiology and pathophysiology with high fidelity, which is suitable for drug development applications [66]. These studies support the potential value of microfluidic reproductive models not only for embryo culture but also for mechanistic investigation and safety assessment.

However, significant challenges remain for translation to clinical and pharmaceutical applications. Only a limited number of reproductive microfluidic technologies have progressed to clinical validation, with key barriers including the need for long-term clinical outcome data, standardization of results across various patient populations, and regulatory challenges and restriction over fabrication material [31,67,68]. Before translation to human embryo culture, the platform would require optimization of flow conditions, shear stress, chamber geometry, medium turnover, and material compatibility, including replacement of PDMS with more embryo-compatible material or validated PDMS surface-treatment strategies. In addition, larger validation studies should include detailed embryo quality assessment beyond morphology and comply with ethical and regulatory requirements for human embryo research.

Several limitations of the present study should be acknowledged. First, this study utilized mouse embryos rather than human embryos, which limits direct clinical translation. Second, NIH/3T3 fibroblasts were used as functional helper cells rather than primary endometrial or oviductal cells, and therefore may not fully reproduce the physiological embryo-reproductive tract microenvironment. Third, the sample size was small, with limited embryo number and donor-level replication. Therefore, no formal a priori power analysis or inferential statistical testing was performed. In addition, embryo quality and viability were assessed only by morphology-based developmental evaluation. However, it could not fully represent subsequent implantation potential. Comprehensive assessments including detailed cellular or molecular analyses, such as total cell number, ICM/TE ratio, apoptosis, oxidative stress, or developmental marker expression, were not performed. Although embryos obtained from the same donor female were distributed across the four culture conditions whenever possible to reduce donor-related variability, formal randomization and blinding were not performed; therefore, allocation bias and observer bias cannot be fully excluded. Finally, shear stress, physicochemical microenvironmental parameters, PDMS-associated adsorption and batch-to-batch chip fabrication reproducibility was not directly quantified. The observed blastocyst formation rates should therefore be interpreted as exploratory feasibility outcomes rather than definitive evidence of efficacy or superiority over conventional culture. These limitations indicate the need for further refinement and validation of the current prototype rather than a limitation of the microfluidic co-culture concept itself. Future validation studies should therefore combine larger randomized and blinded embryo experiments, detailed cellular or molecular quality assessment, quantitative flow and microenvironmental characterization, chip fabrication quality control, material refinement and more physiologically relevant reproductive tract-derived helper cells.

## 4. Materials and Methods

### 4.1. Rationale and Experimental Overview

**Figure 4:**
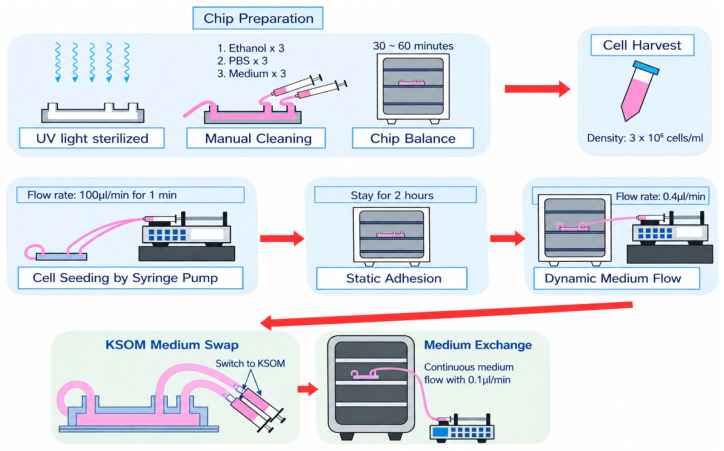
Establishment of the microfluidic chip-based co-culture system for embryo culture. Microfluidic chips were sterilized by UV exposure, sequentially washed with ethanol, PBS, and culture medium, and equilibrated for 30–60 min before use. Helper cells were harvested at a density of 3 × 10^6^ cells/mL and seeded into the chip at 100 μL/min for 1 min, followed by 2 h of static adhesion. Dynamic medium with DMEM flow was then initiated at 0.4 μL/min. During embryo culture, the medium was changed to KSOM and continuously perfused at 0.1 μL/min.

To establish an in vitro model designed to approximate selected features of a uterus-like microenvironment for embryo co-culture, a stepwise experimental strategy was adopted, consistent with recommendations for validating microfluidic cell culture systems [21]. The protocol for processing dynamic microfluidic chips is illustrated in Figure 4. A detailed operational workflow, including cell seeding, embryo trapping, embryo positioning and medium exchange, is provided in Appendix B to clarify the sequential operation of the chip. Mouse fibroblast (NIH/3T3) helper cells were selected as the initial supporting cell type to construct a stable and reproducible co-culture platform, and they have been shown to improve embryo development in co-culture systems [60]. This fibroblast-based architecture served as a foundational model, which may be extended in future studies to develop autologous endometrium-on-chip systems.

The microfluidic chip was fabricated using polydimethylsiloxane (PDMS) as the structural material, and mouse fibroblast cells were employed as co-culture cells. Prior to embryo co-culture, it was necessary to evaluate whether the PDMS substrate and fluidic conditions could support adequate attachment, proliferation, and survival of co-culture cells. If supporting cells were unable to maintain stable growth on the chip, the system would not constitute a suitable in vivo-like microenvironment.

Accordingly, the experimental design consisted of three sequential components:

1. Assessment of fibroblast growth under different culture configurations;
2. Evaluation of the effects of embryo culture medium (KSOM) on co-culture cell viability;
3. Analysis of embryo development under co-culture and control conditions.

### 4.2. Mouse Fibroblast Helper-Cell Culture

Mouse fibroblast cells (NIH/3T3), obtained from the American Type Culture Collection (ATCC), were selected as functional co-culture cells in our microfluidic system experiments. Cells were cultured in a humidified incubator at 37 °C with 5% CO_2_. The growth medium consisted of 75% Dulbecco’s Modified Eagle’s Medium (DMEM) (Gibco BRL, Grand Island, NY, USA) and 25% MCDB 105 medium (Sigma, St. Louis, MO, USA), with antibiotic (penicillin 100 U/mL and streptomycin 100 U/mL), insulin (5 μg/mL), supplemented with 10% fetal bovine serum (FBS) (Invitrogen, Paisley, UK), and 10% charcoal-stripped FBS (Gibco, USA).

For subculture, spent medium was aspirated, and cells were washed with Dulbecco’s phosphate-buffered saline (DPBS, pH 7.0–7.4). Cells were detached using trypsin–EDTA at 37 °C for 3 min, neutralized with serum-containing medium, and collected by centrifugation at 1000 rpm for 5 min. The cell pellet was resuspended in culture medium at the desired concentration for subsequent experiments.

Cell culture procedures were performed according to established ATCC protocols with minor modifications as required for microfluidic experiments. Randomization was not performed in this study. Blinding was not applied during the conduct of the experiments or outcome assessment.

### 4.3. Embryo Collection and Control Culture

All animal procedures were approved by the Chang Gung Memorial Hospital Animal Care and Use Committee (approval number: 2020121412) and were conducted in accordance with institutional guidelines. Female Institute of Cancer Research (ICR) mice (6 weeks old) were intraperitoneally injected with 5 IU pregnant mare serum gonadotropin (PMSG). After 48 h, mice received 5 IU human chorionic gonadotropin (hCG) and were immediately housed with 6–8 weeks old male mice.

Thirty-six hours after hCG injection, successful mating was confirmed by the presence of a vaginal plug, and only plug-positive females were sacrificed for embryo collection. Oviducts were dissected and placed in Petri dishes containing human tubal fluid (HTF) medium. Zygotes were released by gently dissecting the oviducts. Detailed information is listed in Appendix C.

For the control culture, embryos were transferred to potassium simplex optimized medium (KSOM) and cultured in 24-well plates at 37 °C under 5% CO_2_ in a humidified incubator. Embryos introduced into the microfluidic co-culture chip made up the study group. Superovulation, embryo collection, and control embryo culture were conducted following standard and previously described procedures [69].

### 4.4. Microfluidic Chip Design and Fabrication

#### 4.4.1. Chip Architecture

The microfluidic chip consisted of two functional layers.

The upper layer contained the culture chambers, while the lower layer was designed for embryo loading and controlled transport into the culture chambers. The upper main culture chamber, measuring 12 mm in width and 10 mm in height, was divided into three interconnected compartments, allowing fluid exchange while preventing cell loss through interconnecting microchannels (width 100 μm). Meanwhile a single outlet on the opposite side served as the exit for metabolic waste.

The lower layer enabled embryo loading, positioning, and transfer into the culture chambers with connecting microchannels. Flow resistance structures were incorporated to confine embryos within the designated culture chambers. The inlet and outlet ports were all 6 mm in diameter.

#### 4.4.2. Mold Fabrication and PDMS Casting

Silicon wafers were coated with SU-8 photoresist using a spin coater at 1000 rpm to reach the chip height average at 220 μm. Wafers were subjected to soft baking on a 65 °C heating plate where the temperature was slowly raised to 95 °C and held for 50 min, followed by ultraviolet exposure through a photomask, post-exposure baking, and development to form the mold pattern.

PDMS prepolymer and curing agent were mixed at a 10:1 ratio, degassed under vacuum, and poured onto the mold. The PDMS was cured at 90 °C for 30 min, peeled from the mold, and cut into individual chips.

The PDMS layers were bonded to glass substrates or additional PDMS layers using oxygen plasma treatment to enhance surface helper-cell bonding strength.

### 4.5. Static and Dynamic Culture of Supporting Cells on the Chip

Fibroblast cells were prepared at a density of 3 × 10^6^ cells/mL, and 100 μL was injected into the microfluidic chip to evaluate the compatibility of the PDMS substrate with co-culture cell growth.

#### 4.5.1. Static Chip Culture

In the static chip group, fibroblast cell suspension was injected into the chip, and the system was maintained without continuous flow. Culture medium 100 μL was manually replaced every 24 h. Cell attachment, morphology and surface coverage were monitored microscopically at 24 h intervals for up to 72 h.

#### 4.5.2. Dynamic Chip Culture

In the dynamic chip group, fibroblast cells were injected into the chip and allowed to adhere to the PDMS surface during a 2 h static incubation period. Subsequently, dynamic medium perfusion was initiated at a flow rate of approximately 0.4 μL/min with DMEM via the inlet. This flow rate was selected to provide continuous nutrient supply while minimizing shear stress on attached cells. Cell attachment, morphology and surface coverage were monitored microscopically at 24 h intervals for up to 72 h.

### 4.6. KSOM Transition and Co-Culture Cell Viability Assessment

Because the culture requirements for supporting cells and embryos differ, the effect of embryo culture medium (KSOM) on fibroblast viability was evaluated. Fibroblast cells were cultured for 48 h until surface coverage exceeded 80%, after which the original culture medium was replaced with KSOM.

Three experimental conditions were examined:

1. Fibroblast cells cultured in 24-well plates without medium replacement;
2. Fibroblast cells in 24-well plates with manual KSOM replacement;
3. Fibroblast cells in the microfluidic chip with dynamic KSOM perfusion.

In the dynamic chip, KSOM was introduced at a flow rate of approximately 10 μL/min after the establishment of stable cell attachment, followed by continuous perfusion at 0.1 μL/min to maintain nutrient supply. Cell viability was assessed using LIVE/DEAD™ Viability/Cytotoxicity Kit (Invitrogen, Paisley, UK) fluorescence staining according to the manufacturer’s instructions.

Briefly, cells were incubated for 30 min at 37 °C, followed by fluorescence microscopy imaging. Viability was calculated as the percentage of calcein-positive cells relative to total cells.

### 4.7. Embryo Co-Culture Experiments

For embryo co-culture, dynamic microfluidic chips containing established fibroblast cell layers (>80% confluence, 48 h post-seeding) were used. Embryos were introduced into the chip and cultured under continuous, low-flow perfusion at 0.1 μL/min with KSOM medium.

Embryo development was recorded based on morphological evaluation, including cleavage stage progression, morula formation, blastocyst formation and developmental arrest, or degeneration. Embryonic development stages, from the 8-cell stage to the blastocyst stage, were observed at intervals of 24 h up to 72 h under four different culture conditions.

The primary endpoints were blastocyst formation rate and developmental arrest rate.

Four experimental groups were compared:

1. Control group: Embryos cultured in conventional 24-well plates with KSOM (*n* = 9 embryos);
2. Static chip group: Embryos cultured in microfluidic chips with co-culture cells but without dynamic perfusion (*n* = 6 embryos);
3. Dynamic co-culture group: Embryos cultured in microfluidic chips with fibroblast co-culture and continuous perfusion (*n* = 9 embryos);
4. Dynamic group without co-culture cells: Embryos cultured in microfluidic chips with continuous perfusion (*n* = 9 embryos).

The reported *n* values indicate the number of embryos included in the final developmental analysis for each culture condition. Embryos obtained from the same donor female were distributed across the four culture conditions whenever possible to reduce donor-related variability. Within each culture condition, embryos cultured in different chips were derived from different donor females, providing donor-level replication across chips. No formal a priori power analysis was performed because the present study was designed as a feasibility and proof-of-concept study rather than a powered efficacy comparison. Because of the limited embryo number and feasibility-oriented study design, no formal inferential statistical testing was performed for embryo developmental outcomes. Results were therefore reported descriptively as n/N and percentages.

## 5. Conclusions

We developed a microfluidic embryo co-culture platform that integrates compartmentalized cell culture with controlled dynamic perfusion with the aim of approximating selected cellular and dynamic features of the uterine microenvironment. The system supported fibroblast growth under static and dynamic conditions, maintained cell viability following KSOM medium transition, and enabled mouse embryo development to the blastocyst stage. The observed blastocyst formation proportions represent descriptive feasibility outcomes and do not establish superiority or causal effects among tested culture conditions.

This platform provides a technical framework for integrating helper-cell co-culture, dynamic medium exchange, and embryo development within a compartmentalized microfluidic platform. The modular architecture provides a foundation for future development of patient-specific co-culture systems using iPSC-derived endometrial cells, disease modeling applications, and reproductive toxicology screening. Optimization of flow parameters, material compatibility, and further validation with human embryos and endometrial cells will be necessary to establish clinical utility.

## Figures and Tables

**Table 1:** Blastocyst formation rates at 72 h under different embryo culture conditions.

Culture Condition	Blastocyst Formation Rate at 72nd Hour (%)	*n*/*N*
24-well plate co-culture	100.0%	9/9
Dynamic microfluidic with co-culture	55.5%	5/9
Dynamic microfluidic without co-culture	33.3%	3/9
Static chip culture with co-culture	0.0%	0/6

Data are presented as the percentage of embryos reaching the blastocyst stage at 72 h, with the corresponding number of blastocysts over the total number of embryos cultured in each group (*n*/*N*, blastocysts/embryos).

**Figure A4:**
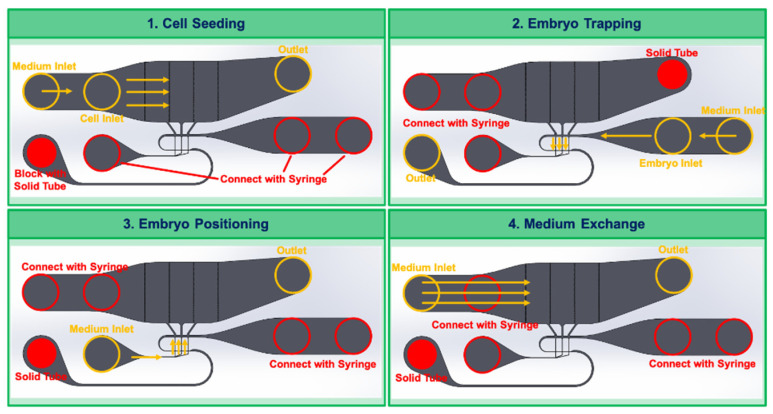
Operational workflow of the microfluidic embryo co-culture chip. The workflow illustrates the sequential operation of the chip during embryo co-culture. (**1**) Cell seeding: Helper cells were introduced into the upper co-culture chamber through the cell inlet, while culture medium was supplied through the medium inlet to guide cell distribution and attachment. Ports in the lower embryo handling region were temporarily blocked or connected to syringes to prevent unintended cell dispersion into the embryo loading region. (**2**) Embryo trapping: After sufficient helper-cell attachment and growth, embryos were introduced through the embryo inlet in the lower layer and guided toward the trapping region, where flow-resistance structures enabled embryo separation and temporary retention. (**3**) Embryo positioning: Embryos were subsequently transferred from the trapping region into the designated co-culture chamber through controlled medium flow via the embryo positioning inlet. (**4**) Medium exchange: After embryo positioning, dynamic medium perfusion was resumed to maintain nutrient supply and metabolic waste removal during embryo–helper-cell co-culture. In all panels, yellow arrows indicate the direction of medium flow.

## Data Availability

The original contributions presented in this study are included in the article. Further inquiries can be directed to the corresponding authors.

## Abbreviations

The following abbreviations are used in this manuscript:

ART	Assisted reproductive technologies
ATCC	American Type Culture Collection
DMEM	Dulbecco’s Modified Eagles Medium
DPBS	Dulbecco’s phosphate-buffered saline
FBS	Fetal bovine serum
hCG	Human chorionic gonadotropin
HTF	Human tubal fluid
ICR	Institute of Cancer Research
KSOM	Potassium simplex optimized medium
MPS	Microphysiologic systems
NIH/3T3	National Institutes of Health 3-day transfer mouse fibroblast
PDMS	Polydimethylsiloxane
PMSG	Pregnant mare serum gonadotropin

## Appendix A. Full Developmental Trajectories of Embryos Cultured Under Different Conditions

Embryos were followed from E1.5 to E4.5 under the indicated culture conditions. M. indicates morula and B.C indicates blastocyst. Morulae were identified by compacted blastomeres without a visible blastocoel cavity, whereas blastocysts were identified by the presence of a visible blastocoel cavity. Earlier cleavage-stage embryos were labeled according to cell number where applicable. Scale bar = 100 μm.

## Appendix C. Animal Information and Reporting Details

### Appendix C.1. Animals

Outbred ICR mice were obtained from BioLASCO, Taipei, Taiwan. Female mice were 6 weeks old and male mice were 6–8 weeks old at the time of mating. Animals were maintained under standard husbandry conditions (12 h light/dark cycle, 22 ± 2 °C, ad libitum access to food and water) in the institutional animal research center.

### Appendix C.2. Mating and Embryo Collection

Female mice were paired with males overnight, and the presence of a vaginal plug the following morning was used to confirm successful mating. Only plug-positive females were included for embryo collection. Thirty-six hours after hCG injection, plug-positive females were euthanized by CO_2_ inhalation followed by cervical dislocation. Oviducts were dissected and placed in human tubal fluid (HTF) medium, and zygotes were released by gently tearing the ampulla.

### Appendix C.3. Scope of Animal Use

The approved animal protocol covered both the embryo culture experiments reported in the present study and additional downstream implantation-related procedures within the broader research project. The present manuscript includes only mating, embryo collection, and in vitro culture experiments; accordingly, only animals directly relevant to these procedures are reported.

### Appendix C.4. Sample Size Considerations

Sample size was determined empirically based on expected embryo yield per successfully mated female and the requirement for repeated experiments to compare the conventional culture dish group with the microfluidic chip co-culture group. Because embryo developmental rates may vary among donor females, repeated experiments were performed to ensure sufficient analyzable data.
